# Nurses' experience of work stress related to COVID‐19 regular prevention and control in China: A qualitative study

**DOI:** 10.1111/jonm.13528

**Published:** 2021-12-21

**Authors:** Zhaobin Jiang, Shengnan Wang, Zhengfu Shen, Xiaoyan Zhao, Fuzhi Wang, Yongxia Chen, Yan Qiao, Tao Wei, Pingping Dong, Sanqing Ding, Xiumu Yang

**Affiliations:** ^1^ Faculty of Nursing Bengbu Medical College Bengbu China; ^2^ General Office North Anhui Health Vocational College Suzhou China; ^3^ Research Center for General Practice Education Development of Bengbu Medical College Anhui Province Humanities and Social Science Key Research Base Bengbu China; ^4^ School of Health Management Bengbu Medical College Bengbu China; ^5^ Nursing Department The First Affiliated Hospital of Bengbu Medical College Bengbu China; ^6^ Infectious Disease Department The First Affiliated Hospital of Bengbu Medical College Bengbu China; ^7^ School of Public Policy and Management China University of Mining and Technology Xuzhou China

**Keywords:** COVID‐19, nurse, qualitative research, regular epidemic prevention and control, work stress

## Abstract

**Aim:**

To explore the experiences of nurses' work stress related to COVID‐19 regular epidemic prevention and control in China.

**Background:**

The global COVID‐19 epidemic is still severe, and China's ongoing regular epidemic prevention and control still cannot be relaxed, which places demands on nurses.

**Methods:**

Thirty nurses and eight nurse managers were interviewed using semistructured in‐depth interviews, and the data were analysed by the Colaizzi seven‐step analysis method.

**Results:**

Four themes were extracted as follows: environmental factors, organizational factors, personal factors and positive factors in coping with stress.

**Conclusions:**

Nursing managers should pay attention to construction of the first‐line departments of regular epidemic prevention and control. The shortage of nurses' human resources and the increase of nurse–patient conflicts are problems that need to be solved urgently. In addition, this research also emphasizes the importance of promoting nurses' stress‐related growth and thinking about the possibility of reform.

**Implications for Nursing Management:**

The construction of the hospital environment and increasing the resilience of nursing teams require attention. We should attach importance to the training of nurses' communication skills and provide sufficient organizational support and economic guarantees for nurses. Finally, perhaps we should also consider whether it is necessary to reform the relevant hospital systems and how to reform them.

## 1 BACKGROUND

According to the information provided by the WHO, as of 13 October 2021, there were 238,521,855 confirmed cases of COVID‐19 worldwide, 4,863,818 deaths and more than 300,000 newly confirmed cases per day (WHO, 2021). The prevention and control of the epidemic is far from over, and we still need to attach great importance to it. Since the outbreak of the COVID‐19 epidemic, hospitals have remained on the front line of prevention and control, and all medical staff have been under great pressure. As the largest group among medical staff and the closest contact with patients, nurses deserve our attention. Murat et al. (2021) found that nurses suffered high levels of stress and burnout and moderate depression during the outbreak of the epidemic in Turkey. Shahrour and Dardas (2020) found that 64% of nurses experienced acute stress disorder, and 41% of nurses had psychological distress during the outbreak of the epidemic in Jordan. A meta‐analysis of the literature on the mental health status of front‐line medical staff published between December 2019 and June 2020 showed that the incidence of depression among front‐line nurses who participated in caring for COVID‐19 patients during the outbreak of COVID‐19 was 28%, and the incidence of anxiety was 22.8% (Salari et al., 2020). Since April 2020, the epidemic in China has been well controlled, and China has entered the stage of regular prevention and control (Zheng et al., 2020). There have been many studies investigating the work stress and mental health of nurses during the outbreak period (An et al., 2020; Tu et al., 2020; Zhan et al., 2020; Zhang, Miao, et al., 2020), but there were few studies on the work stress of nurses during the regular prevention and control period. Judging from previous experience, nurses' work stress during the regular prevention and control period must be much less than that during the outbreak period. However, Wu et al. (2020) investigated the incidence of burnout of front‐line nurses and nurses in general wards during the outbreak period and found that nurses in general wards were more prone to burnout. This shows, on the one hand, that we may underestimate the level of work stress that nurses bear during the regular prevention and control period. Therefore, we designed this study to explore the work stress of Chinese nurses during the regular epidemic prevention and control period. Considering that Chinese hospitals have taken a series of measures to address regular epidemic prevention and control and that the working environment of nurses has changed greatly, the previous scale for measuring work stress may not be applicable to this study. Therefore, this study adopted the phenomenological approach in qualitative research to explore the work stress experience of nurses related to regular epidemic prevention and control. The global COVID‐19 epidemic is still severe, and the findings of this study may provide some references for other countries to respond to the epidemic in the future.

## 2 METHODS

This study is qualitative. A phenomenological approach was used to explore the theme of nurses' work stress experience during COVID‐19 epidemic regular prevention and control. And this study was conducted in January 2021.

### 2.1 Theoretical framework

Robbins' occupational stress model (Fradreck, 2018; Humayon, 2018) was used as the theoretical framework of this study. The stress model identified three potential stressors: environment, organization and individual. Based on this, a semistructured interview outline of nurses' work stress experiences during regular epidemic prevention and control was compiled.

### 2.2 Participants

The subjects of the study were mainly nurses and head nurses who worked in the clinic during the regular prevention and control period in two hospitals in East China and North China. The sampling methods were convenience sampling and snowball sampling. The inclusion criteria were as follows: (a) having a nurse qualification certificate, (b) participating in clinical work for ≥3 months during the regular epidemic prevention and control period and (c) being willing to participate in this study. Nurses and head nurses from various hospitals and multiple departments in the same hospital were selected to ensure the adequacy of the samples. The sample size was ultimately determined by information saturation.

### 2.3 Data collection

The interview methods were face‐to‐face interviews and WeChat voice interviews. Face‐to‐face interviews were conducted in the nurses' free time or after work and in quiet lounges or offices. WeChat voice interviews were scheduled with the interviewees in advance and were conducted when the interviewees were resting at home or in other quiet places. Before the interview, the researcher informed the interviewees of the purpose, significance, anonymity and confidentiality of the study. The consent of the interviewee was sought for the recording, and the dissenters only took notes. The duration of each interview was approximately 20–60 min. During the interview, more in‐depth questions or new related topics could be discussed according to the actual situation.

### 2.4 Interview outline

Based on Robbins' occupational stress model (Fradreck, 2018; Humayon, 2018), an interview outline was developed under the guidance of experts with qualitative research experience. After the outline was initially formed, two nurses who participated in clinical work during regular epidemic prevention and control were selected for pre‐interviews, and the interviewees were asked to point out unreasonable problems in the interview outline. The interview outline was modified according to the pre interview results, and the final outline was completed after being reviewed and approved by experts. The outline of the nurse (head nurse) interview is as follows: ① What stress has the regular epidemic prevention and control put on you (nurses)? ② What changes have taken place in the environment of hospitals and departments under regular epidemic prevention and control? ③ What work has regular epidemic prevention and control added to you (nurses)? ④ During regular epidemic prevention and control, which aspects of the work requirements of the leaders (you) are stricter, which make you (nurses) feel stressed? ⑤ What impact does regular epidemic prevention and control have on the interpersonal relationship of nurses (you)? ⑥ Has regular epidemic prevention and control had some impact on the nurse's (your) family? ⑦ Are you (nurses) satisfied with your current salary level? Does regular epidemic prevention and control affect the income level? ⑧ In addition to the above, do you have anything to add about regular epidemic prevention and control?

### 2.5 Data analysis

The recordings were listened to repeatedly, and the recording data were transcribed verbatim. Members of the research team jointly verified the transcribed content. The transcribed text was imported into the qualitative research software NVivo 12.0 plus, and the data were encoded and refined according to Colaizzi's seven‐step analysis method (Colaizzi, 1978).

## 3 RESULTS

A total of 30 nurses and 8 head nurses were interviewed. The basic information of the interviewees is shown in Table 1. Five nurses were nonrecorded, represented by N1‐N5, and the rest were represented by NA‐NY. Ns1‐Ns8 was used to represent the head nurses.

**TABLE 1:** Basic information of interviewees

Attribute	Information
Gender	4 males (10.5%) and 34 females (89.5%)
Age ( $\overset{-}{X}$ ± SD)	22–50 years old (30.16 ± 7.20)
Length of service	3 months to 30 years
Department	Nurses: fever clinic (6), infection department (5), ICU (6), emergency department (1), paediatrics (1), internal medicine (3), surgery (2) and other departments (4)^a^ Head nurses: infection department (3), emergency department (2), respiratory department (2) and ICU(1)^a^

^a^ Figures in brackets indicate the number of interviewees.

Four themes were extracted as follows: environmental factors, organizational factors, personal factors and positive factors in coping with stress. The first three themes were described under the framework of Robbins' stress model, which not only described the stressors of regular epidemic prevention and control but also described the stress experience of nurses. The last theme described the positive psychological experience of nurses in coping with the stress brought by regular epidemic prevention and control.

### 3.1 Environment factors

#### 3.1.1 Technical factors

The detection of novel coronavirus involves nucleic acid detection technology, which takes a long time from throat swab collection to nucleic acid test results. In the process of waiting for the results of nucleic acid detection, patients may conflict with medical staff due to lack of desired treatment and resistance to isolation measures.

During that time, many urgent patients were admitted to the emergency department. However, as soon as they had a fever, they were transferred to the fever clinic, and the patient's family members were very anxious. At that time, the nucleic acid results took 24 h. Before December, it took 1–2 days for the patients admitted to the emergency ward to be transferred to other departments. (NF)

Fever clinics only exclude COVID‐19 infection and do not perform any treatment. Nucleic acid results can now be completed sooner than before, with results said to be available within 2 h. However, it takes at least 3 or 4 h for the results to be available. Thus, patients just sit and wait, leading them to be very impatient and resulting in substantial friction. (NG)

The nucleic acid test takes too long, and the patient loses patience. (N2)

Due to the long time required for nucleic acid testing, nurses were also in a state of tension while accompanying patients waiting for results.

All patients are unknown. We do not know why he has a fever. We still have some fear. After all, the epidemic is still serious. (NC)

The results of the nucleic acid will not be known until tonight, but with this patient under our supervision all day, there must be pressure on your mind. (NI)

#### 3.1.2 Hospital environmental factors

As fever clinics and infection departments have undertaken the task of isolation, the resettlement site was far from the core hospital area of the hospital, and channel management was relatively strict during regular epidemic prevention and control. It often took substantial time for fever patients to find the ward. Some patients were dissatisfied with this and vented their emotions on the medical staff.

The location of the fever clinic is reasonable; that is, it is an infected building that is far away from the entire hospital area. It is unreasonable that it is too far away for patients to find. (NC)

The patient took a long time to get here from the clinic…he is not angry over there, he is just angry right here, because he is facing you now. (NG)

The hospital implemented a ‘semiclosed’ management mode for patients and their families. All channels of the hospital were strictly managed, and most channels in the ward were managed by the ward itself. Some wards had no access control, and the channel management depended on nurses, which increased the workload of nurses and depleted the human resources of nurses.

The management of each floor depends entirely on our medical staff. We have many other things to do; how can we take care of so many…(Ns3)

From six o'clock in the morning to nine thirty in the evening, the gate is guarded, which is equivalent to assigning three nurses to guard every day. (Ns4)

We hope it has a system or access control so that there is no need to artificially block the patient's family members, resulting in unnecessary disputes…because the labour cost of nurses is actually quite large.(Ns5)

### 3.2 Organizational factors

#### 3.2.1 Workload increase

Regular epidemic prevention and control has added much work to nurses, and some changes have taken place in the daily work processes of nurses. This additional work includes the treatment process of fever patients and patients from high‐risk areas, admission of new patients, increasing the daily work of nurses in the ward. The work content has increased, but there is no more nursing human resources, which makes nurses busier and more stressed.

For some patients who return from medium‐ and high‐risk areas or have fever of unknown cause, the treatment procedure is much more complicated than before. Our nurse should first guide the patient to the infection department and then go back to the emergency department to give him normal treatment. If the patient cannot be checked, he has to be isolated in a single room. (NW)

After the nucleic acid results were obtained, they were admitted to the hospital. As a result, some patients can only come at night. The procedure of receiving new patients is more complex, and sometimes, the patient's condition is more complicated to address. There are only two nurses working at night, and we still have a lot of routine work to do, so we would be very busy and stressed. (NO)

Nurses should not only perform the nursing routine of the ward but also verify ‘one patient, one companion’ escort certificate and infection control management. The workload of clinical nurses has increased. (Ns3)

#### 3.2.2 Increased work difficulty

One of the key points of epidemic prevention and control is to reduce personnel gathering. Due to China's cultural habits, many patients' families and relatives and friends who come to visit patients often gather in hospitals, which increases the risk of epidemic transmission. During the regular epidemic prevention and control period, hospitals prohibited visits and took measures to limit the number of patients' family members present. For each patient, only one family member is allowed to accompany them in the ward, and the family member must also provide proof of a negative nucleic acid test result and cannot enter or exit at will. Many patients and their families cannot understand or disapprove of this. It is difficult for nurses who have the most contact with patients and their families to explain and communicate.

Now, the requirement is ‘one patient one companion’, and the family members also require nucleic acid testing. Sometimes some patient's family members do not understand and quarrel with us, saying that the patient's condition is serious and that the patient needs more escort…(NX)

Regardless of how strict your management is, there will always be some family members slipping into the ward. If you want to let him go, you basically have to talk to him for a long time…For the satisfaction survey, the satisfaction with a required escort is certainly not high. (NE)

It is difficult for nurses who are on duty at the door of the ward to prevent patients' families from entering the ward.

We told him that this is an isolation ward. You cannot go out; you should wear a mask. Then, the patient said, where is the epidemic now? I watched the news, nothing happened. (Ns2)

Some patients are not very conscious and sometimes do not wear masks. We have to remind them to wear masks all the time. In addition, patients are now required not to leave, but some patients will leave or go to the cafeteria to buy food and something else. It is difficult to keep them from going out for 24 hours. (NQ)

#### 3.2.3 Increased role stress of nurses

Regular epidemic prevention and control has increased the workload and difficulty of work, which increases the stress of nurses' work tasks. At the same time, hospitals have also put forward higher requirements for nurses, which has also increased the stress associated with nurses' role tasks.

First, training, inspection and assessment related to regular epidemic prevention and control have increased.

There are a lot of things to learn, such as the meetings…(NJ)

More inspections…such as the inspection of hospital sense control. There are also regular training, examinations, and then irregular casual visits about your work status…(NB)

After the night shift, we have to listen to some lessons on epidemic prevention and control…several times a week. (NN)

The requirements are stricter…more inspection items…for example, if the door of your ward is not locked in time or a patient is accompanied by two family members or one family member but not the fixed one…they would check all of these things. (NP)

Second, with the implementation of regular epidemic prevention and control, hospitals overly rely on nurses. Nurses undertake most of the regular epidemic prevention and control work in the hospitals, and some nurses may feel unbalanced.

The guarding and other things are all undertaken by nurses. Nurses take on more work, doctors do not have to…may have some emotions. (NO)

Nurses are the main force in regular prevention and control. (Ns4).

### 3.3 Personal factors

#### 3.3.1 Decrease in revenue

Due to the impact of the epidemic environment and strict prevention and control of hospitals, some patients choose not to see a doctor temporarily or not to see a doctor in hospitals with strict prevention and control. The number of patients in the hospital has decreased, the income of the hospital has been reduced and nurses have also been affected.

The patient is affected. Our hospital is a designated hospital for the epidemic situation. Patients have to register a lot of information when admitted, and some have to scan the code…(NI)

Nucleic acid testing was slow at the beginning. Some patients have to wait until the next day to be admitted to the hospital; some just do not stay in the hospital at all, some go to other hospitals and the overall flow of people in the department is reduced. (NJ)

#### 3.3.2 Limitation of daily life

The prevention and control of epidemics require avoiding personnel gathering as much as possible, so the hospital has required employees not to participate in gathering activities. In addition, to control the flow of personnel, it has stipulated that if employees go to other cities, they need to report such, and when they return, they need to provide a report with a negative nucleic acid test result. For reasons of epidemic prevention and control, nurses' daily lives have been partially limited.

Cannot go out, cannot have dinner or play together…always stay at home…I think it will be a little boring, and I will be in a bad mood. (NJ)

Due to the restrictions of the epidemic, it has become inconvenient for nonlocal nurses to return to their hometowns to visit their family members.

It's troublesome to go back home. (NQ)

The biggest impact is that for my relatives, I pay less, I have no time to go home, I cannot go home…I went home once last year; it's been almost one year…I did not go back last Spring Festival, and I may not be able to go back this year either. (Ns1).

### 3.4 Positive factors for coping with stress

During the interview, we found some positive psychological feelings of nurses outside the framework of Robbins' stress model, which are described here as an extension.

#### 3.4.1 Hope

Although the outbreak of the epidemic and the current regular epidemic prevention and control have brought tremendous pressure to nurses, nurses still firmly believe that the disaster will be overcome, face reality optimistically and are full of hope in their hearts.

2020 was an unfortunate year, with a lot of disasters. But, I asked one nurse that day, I said, what did the little girl gain in 2020? The little girl said, ‘I have never seen high‐flow oxygen inhalation before, and now I can use it skilfully’.(Ns2).

#### 3.4.2 Unity

In the difficult environment of facing the epidemic together, colleagues have become more united and have deeper feelings toward each other.

When there is no epidemic, we may go back to our own homes at lunch. Later, because of epidemic prevention and control, no one would go home at lunch. We all ate together, talked and chatted together. I think we know each other better and communicate more with each other during epidemic prevention and control. (NB)

#### 3.4.3 Patience

During the normalization of epidemic prevention and control, nurses need to assist hospitals in personnel control and to explain the importance and necessity of nucleic acid testing to patients and their families. The incomprehension and noncooperation of patients and their families is one of the biggest work stressors for nurses during the regular epidemic prevention and control period. In the face of this situation, nurses move patients through full patience and sincere communication so that patients can understand and support the work of the hospital.

You could give him a little more explanation…telling him not to charge him, and it is not a painful operation…if you communicate patiently, most patients can understand you. (NI)

#### 3.4.4 A sense of security

Although the measures taken by the hospital in response to normalized prevention and control have created some pressure, it also made nurses feel safe and secure and made nurses believe that the epidemic is preventable and controllable; thus, their hearts are more stable.

In fact, we are not as nervous at work as when the epidemic first broke out because we think it can be controlled. (NB)

Hospital infection control is stricter. I think it's good to be stricter. It's more secure. (NE)

## 4 DISCUSSION

Among all COVID‐19 test methods, the antibody test takes the least time, less than 20 min to obtain test results. However, antibody tests cannot provide direct diagnostic evidence and cannot detect early infection, so they are applicable only for screening and auxiliary diagnosis (Carbonell‐Sahuquillo et al., 2021). The nucleic acid test is still the most commonly used COVID‐19 test method and can provide direct diagnostic evidence, and the test process takes several hours (Sule & Oluwayelu, 2020; Yüce et al., 2021). Due to technical limitations, it is difficult to significantly shorten the test time of COVID‐19 in the short term. The nurse–patient conflicts caused by patients waiting for nucleic acid test results can be solved only from the perspective of management. Our interviews found that such conflicts between nurses and patients mostly occur in fever clinics, and most of them are caused by a lack of desired treatment and the emotional excitement of patients. To solve such problems, fever clinics should be able to meet some needs of patients and take appropriate measures for patients' diseases. If it is impossible to deal with them, it should be carefully explained to patients and their families to make them feel at ease. As the first‐line of defence of hospital departments, fever clinics play a vital role during the outbreak of the COVID‐19 epidemic in China (Wang et al., 2021). Managers should also pay attention to the construction of fever clinics during regular epidemic prevention and control.

Experience during the outbreak period tells us that a reasonable hospital layout and complete hospital facilities can improve work efficiency and prevention and control effects (Chen et al., 2020; Lai et al., 2020). The problem of ward distance cannot be solved from the perspective of architecture, but patients can be guided to find the ward quickly with clear route instructions by setting up road signs and hospital layout maps. Our interviews found that many nurses held the hope that the epidemic could end soon and that the hospital could restore the previous order as soon as possible. Although nurses complained about the ‘temporary’ measures that required nurses to guard the ward gate due to the lack of access control of the hospital, they could understand and cooperate. What managers should consider is that perhaps we should not restore the previous order but take this opportunity to establish a new order. The prevention and control of COVID‐19 has consumed a lot of our energy, which is a challenge for us, but at the same time, it is also an opportunity for us to establish a new medical management system order and comprehensively improve hospitals' abilities to respond to public health emergencies. Should these ‘temporary’ measures of regular epidemic prevention and control, such as the management of patients and their families and the control of hospital infections, be upgraded to ‘permanent’ measures? Should the hospital‐related management system reform? This is a question worth pondering.

The increase in workload has made nurses' human resources more strained. Recruiting more nursing staff is the most direct solution; however, even before the outbreak, the human resources of nurses were in short supply worldwide. No more nurses could be recruited (Shaffer et al., 2020). Some scholars (Duncan, 2020) believe that means of improving the resilience of nursing teams and make limited nurse human resources play a greater role is an issue that we should seriously consider in the current epidemic. As early as the beginning of this century, nursing managers of some hospitals in China advocated a method to increase the resilience of the nursing team, that is, establishing ‘mobile nurse banks’, and formulated detailed methods for training and managing mobile nurses (Wang et al., 2005). According to the reports of hospitals (Han et al., 2020; Wang et al., 2005; Wu, 2021; Ye et al., 2011) that have established mobile nurse banks, ‘mobile nurses’ have played an important role in fighting against SARS, avian influenza A (H1N1) and the current COVID‐19 epidemic and have buffed the pressure of nurse human resource shortages caused by various emergency and nonemergency situations for hospitals during nonepidemic periods. Unfortunately, only a few hospitals have established mobile nurse banks. Nursing managers may consider establishing mobile nurse banks to alleviate the shortage of nurse human resources.

Effective nurse–patient communication can improve patient satisfaction and avoid nurse–patient conflicts (Baldwin & Spears, 2019; Lotfi et al., 2019). Our interviews also found that nurses' patient communication can enable some patients to understand and cooperate with the hospital's prevention and control work. Patients' incomprehension and noncooperation of prevention and control measures are the main reasons for the increased difficulty of nurses' work. Therefore, managers can train nurses in targeted communication skills so that nurses can communicate more effectively with patients and can let more patients accept prevention and control measures to improve nurses' self‐efficacy and reduce nurses' stress. Organizational support can improve nurses' psychological resilience and maintain nurses' mental health levels (Carmassi et al., 2020; Cooper et al., 2020; Foster et al., 2020). Income level is positively correlated with job satisfaction and negatively correlated with burnout and turnover (Ran et al., 2020; Wubetie et al., 2020; Zhang, Wei, et al., 2020). During the regular epidemic prevention and control period, while hospitals and managers put forward high standards and strict requirements for nurses, they should also pay attention to providing nurses with sufficient organizational support and economic security and pay attention to nurses' mental health.

Positive factors of nurses in coping with stress related to prevention and control that include ‘hope’, ‘unity’, ‘patience’ and ‘sense of security’ are forms of stress‐related growth and are protective factors against the adverse effects of stress (Yıldırım & Arslan, 2021). Research shows that (Antebi‐Gruszka et al., 2021) positive reappraisal, social support and emotional expression are all related to greater stress‐related growth. Therefore, managers can promote nurses' stress‐related growth by praising and encouraging nurses, giving nurses enough support and listening to nurses' emotional expressions to better deal with epidemic prevention and control.

In addition, this study found that during regular epidemic prevention and control in China, the management stress of head nurses seems to be greater than that of ordinary nurses. However, since the purpose of this study is to explore the work stress experience of ordinary nurses, there was no more in‐depth exploration of the stress of head nurses, and follow‐up research should pay more attention to the stress of nursing managers.

## 5 CONCLUSIONS

Construction of the first‐line departments such as fever clinics should be valued by hospitals and nursing managers, and sufficient financial and organizational support should be given to nurses participating in the prevention and control work. The shortage of nurses' human resources and the increase of nurse–patient conflicts are problems that need to be solved urgently. In addition, this study also emphasizes the importance of protecting and promoting nurses' stress‐related growth. Finally, a question worth pondering is whether it is necessary to take this opportunity to reform the hospital‐related management system and how to reform it.

## 6 IMPLICATIONS FOR NURSING MANAGEMENT

Our study describes the work stress experience of nurses related to COVID‐19 regular epidemic prevention and control in China, highlighting some problems in nursing management. First, the prevention and control of the epidemic should pay attention to the construction of the hospital environment, especially for first‐line departments such as fever clinics. The equipment and facilities should be fully equipped, and the work process should be optimized to improve patient satisfaction and reduce the occurrence of nurse–patient conflicts. Second, managers should consider how to increase the resilience of the nurse team to better prevent and control the epidemic and deal with other emergencies that may occur. Establishing ‘mobile nurse banks’ may be an effective method. Third, effective communication is very important. Effective communication could enable patients to understand and cooperate with the hospital's regular prevention and control measures and resolve most of the nurse–patient conflicts. This can be achieved through targeted communication skills training. Fourth, providing sufficient organizational support and economic security would contribute to the stability of nurses' work. Finally, for the ongoing ‘temporary’ prevention and control measures, is it necessary to take this opportunity to reform the relevant hospital management system and consider how to reform it? This problem is worth pondering by managers. China is the first country to control the spread of the epidemic. Sharing relevant measures of epidemic prevention and control in Chinese hospitals and nurses' work stress experience is intended to provide some reference for medical and nursing managers in other countries to formulate measures to deal with the epidemic.

## CONFLICT OF INTEREST

The authors declare that there are no conflicts of interest.

## ETHICS STATEMENT

Ethical approval was granted by the Research Ethics Committee of Bengbu Medical College (2021‐075).

## AUTHOR CONTRIBUTIONS

Xiumu Yang, Zhengfu Shen, Sanqing Ding, Fuzhi Wang and Xiaoyan Zhao provided research ideas, determined the research theme and designed the research. Zhaobin Jiang, Shengnan Wang, Yongxia Chen and Yan Qiao collected data. Zhaobin Jiang, Shengnan Wang and Pingping Dong analysed the data. Zhaobin Jiang and Shengnan Wang wrote the manuscript. Tao Wei and Pingping Dong translated the article. All authors approved the final version for submission.

## Data Availability

The data that support the findings of this study are available from the corresponding author upon reasonable request.
